# Control strategy for current limitation and maximum capacity utilization of grid connected PV inverter under unbalanced grid conditions

**DOI:** 10.1038/s41598-024-60244-x

**Published:** 2024-05-02

**Authors:** Jyoti Joshi, Vibhu Jately, Peeyush Kala, Abhishek Sharma, Wei Hong Lim, Brian Azzopardi

**Affiliations:** 1 Department of Computer Science and Engineering, Graphic Era Hill University, Dehra Dun, 248002 India; 2 Department of Electrical & Electronics Engineering, School of Engineering, University of Petroleum and Energy Studies, Dehra Dun, 248007 India; 3 Department of Electrical and Electronics Engineering, SRM Institute of Science and Technology, Delhi NCR Campus, Ghaziabad, Uttar Pradesh 201204 India; 4 Department of Computer Science and Engineering, Graphic Era Deemed to Be University, Dehra Dun, 248002 India; 5 Faculty of Engineering, Technology and Built Environment, UCSI University, 56000 Cheras, Kuala Lumpur, Malaysia; 6 The Foundation for Innovation and Research – Malta, 65 Design Centre Level 2, Tower Road, Birkirkara, BKR 4012 Malta; 7 MCAST Energy Research Group, Institute of Engineering and Transport, Malta College of Arts, Science and Technology (MCAST), Main Campus, Corradino Hill, Paola, PLA9032 Malta

**Keywords:** Grid connected PV system, Active and reactive power control, Active power curtailment, Voltage stability, Inverter current limitation, Electrical and electronic engineering, Photovoltaics

## Abstract

Under grid voltage sags, over current protection and exploiting the maximum capacity of the inverter are the two main goals of grid-connected PV inverters. To facilitate low-voltage ride-through (LVRT), it is imperative to ensure that inverter currents are sinusoidal and remain within permissible limits throughout the inverter operation. An improved LVRT control strategy for a two-stage three-phase grid-connected PV system is presented here to address these challenges. To provide over current limitation as well as to ensure maximum exploitation of the inverter capacity, a control strategy is proposed, and performance the strategy is evaluated based on the three generation scenarios on a 2-kW grid connected PV system. An active power curtailment (APC) loop is activated only in high power generation scenario to limit the current’s amplitude below the inverter’s rated current. The superior performance of the proposed strategy is established by comparison with two recent LVRT control strategies. The proposed method not only injects necessary active and reactive power but also minimizes overcurrent with increased exploitation of the inverter’s capacity under unbalanced grid voltage sag.

## Introduction

Distributed generation (DG) got a considerable boost recently^1^. The capacity of distributed generation plants, which primarily comprise photovoltaic or wind-powered units, is relatively high. Among these two, the PV units, are much smaller than the conventional large generation plants. Being smaller, these DG units are scalable, lending them suitable for mass production and lowering the costs, with additional advantages like generation capacity enhancement without the need for investing in new transmission systems, no associated environment degradation, low carbon footprint, and ability of operation over wider voltage range, helpful in keeping the grid stable^2^. With an intention to seek grid support from the non-inertia PV units, all major countries have their own grid codes to specify the permissible range of frequency and voltage during faults^3,4^. These codes, invariably have a desirability of keeping the plants on-grid during transient faults and hold them from tripping when the voltage tends to collapse. This feature termed as low voltage ride through (LVRT) capability is built in the control structure of DG units^5–8^. When the system is stable and operating under normal conditions, i.e., without any fault, harnessing of the maximum yield from the DG PV is achieved using the maximum power point tracking^9–12^.

When a fault (such as a short circuit, flickering, or loss of grid power) occurs on the grid, even if it is transient in nature, the conventional grid-tied PV inverters automatically cut themselves off from the grid. The inverters are configured in this fashion to prevent damage from transients of over current or over voltage. Every inverter linked to such a problematic grid line would have similar trip-offs from the grid. As a result, a significant portion of the distributed generators would cease producing power. Eventually, the net load on the distribution feeder would increase, which would cause a sharp reduction in voltage at PCC. This voltage drop can result in flickering lights and poor power quality at the consumer’s location, which might interfere with digitally controlled appliances and equipment in homes or offices. Customers may lose money due to the malfunctioning equipment, which will lead to complaints to the utility provider. If these conditions persist over time, the local utility may be tempted to discourage more PV systems on the grid. Low-voltage-ride-through (LVRT) technology integrated into a PV system is the obvious solution for this issue. When a transient fault event occurs, the PV inverters with integrated LVRT features will continue serving the grid and avoid unnecessary interruption. In other words, there would be no flashing or other power-related issues with the home equipment. Nevertheless, a well-designed voltage-ride-through unit would make sure that the solar PV system shuts off to protect it from damage in the event of any sustained problem in the grid, such as a persistent short circuit or a loss of power on the grid. Low voltage ride-through (LVRT) capable inverters inject reactive power to help with fault recovery during periods of grid sags in addition to withstanding grid sags^13,14^. The goal of the LVRT inverter is to maintain grid connectivity during transient faults by disabling and de-activating the under/over voltage and over current relays.

Several investigations have been carried out in designing line protection devices and their operation in LV and MV feeders during low-voltage-ride-through period. In^15^, the authors have presented a solution for early operation of the fuse in LV distribution line, by deploying a current limiting device (CLD) near the fuse. The CLD limits the short circuit current so that the fuse could not contravene the LVRT requirements. ^16^ proposes a fault-current-limitation based solution, by employing a CLD which in turn prevents the unintentional islanding situation in GCPV system.

A large increase in distributed generation (DG) on a power system might have an adverse effect on the stability and reliability of the grid, especially during outages. The higher this penetration of DG, more is the adverse effect on stability. Countries that have substantial DG penetration have devised their own Grid codes (GCs) to guide the operators. These code guidelines specify the type of faults that the grid should be able to endure, as well as the procedure that should be followed in such cases. As DG penetration grew, network operators began injecting reactive power into the grid to help maintain grid voltage and prevent voltage collapse.

Power electronic inverters that interface with RESs and the grid are designed to improve quality of power and help the system to remain stable through the disruptions or grid faults of short durations, especially when the grid is unbalanced. To obviate chances of undesirable relay-trips and generation loss, grid-connected inverters must be able to survive sag in the grid voltage and stay connected. In order to prevent voltage collapse, these inverters are required to inject flexible amounts of active and reactive powers to maintain grid voltage^13^.

For process industries, voltage sags constitute the most serious power quality issue. A drop in the RMS voltage during a period of 0.5 to 1 min, often between 0 and 0.9 p.u. is defined as voltage sag. To overcome voltage sags, low-voltage ride-through is vital. As a result, power plants might stay connected to avoid tripping and power generation loss. In the event of a fault, quick detection and a quick action to reduce the ill effects of the fault on all equipment, including the inverter itself and upstream plant, on the grid is necessary^17–19^. All these (quick detection and required reaction) features are built into the LVRT enabled inverters along with a capability to inject reactive power during low voltage period of the fault to fulfil the requirements of revised grid codes.

For injecting active and reactive power to the grid, a reference current corresponding to the demand is required to be generated. A proper strategy for generating this current reference (CRG) in accordance with the applicable grid code is essential^20^. One objective of CRG is to improve the quality of the injected power during normal operation of the grid. This can be easily met using any conventional CRG strategy among average active-reactive control (AARC), instantaneous active-reactive (I ARC), balanced positive sequence control (BPSC), and balanced positive sequence control (BPSC). But for an uninterrupted operation under unbalanced faults on the grid will need improvement and modification of these strategies^21^. Conventional strategies lack the current limiting and voltage support features that are so essential during fault-ride-through operation^22^. Chief objective of the chosen CRG is to provide the necessary support so that the voltage at the point of common coupling (PCC) does not dip below the allowable limit, as also the peak value of the current injected is within the safe limits for the inverter to let an unbalanced fault ride through^23,24^.

The goal of voltage support strategy (VSS) is to offer only reactive power during grid voltage sags. Reactive current has a significant effect on the PCC voltage when the grid is weak. Several authors have proposed various reactive power injection techniques keeping in view the kind of voltage sag^25–27^. In^25^, the authors proposed a current reference control technique that allows for adjustable voltage support. The authors in^26^, showed an improvement of the control strategy proposed in^25^, albeit it was confined to symmetric sags, in which case the voltage at the PCC can be improved if the DG plant is able to produce enough reactive current. A voltage control strategy for different types of voltage sags is presented in^27^ by separately controlling the positive and negative sequence reactive power to mitigate the unbalance in the voltages. A CRG method that reduces oscillations in active and reactive powers is proposed in^28,29^. The response time is improved by using a FOPI (Fractional-order PI) controller rather than a traditional PI, PR controller, to achieve the zero steady-state error in the stationary reference frame.

In^30^, the authors suggested a strategy that successfully controls the value of the peak current by employing the positive–negative sequence control (PNSC) by injecting negative sequence inductive current. The suggested method has the advantage of being able to enforce a specified ampere constraint while executing actual power and current injection that is reactive. This ensures that the peak value of the output current of the converter does not violate the specified. However, the scheme suffers from excessive overshoot in inverter current with active and reactive power oscillations. The authors in^31^, proposed a PNSC based current limitation strategy (CLS) by flexibly controlling the active and reactive current references under unbalanced grid fault. Although the technique limits the injected current to the rated value of the inverter current during disturbances, the maximum capacity of the inverter is not exploited under different generation scenarios.

In^32^, the authors proposed a fully flexible current controller that uses the active power and reactive power’s sequence components. The injection of both the positive and the negative sequences of these powers helps to restrict the peak currents to improve ride through services. It also results in utilization of the maximum capacity of the inverter. However, the control strategy has a significant level of complexity because it is highly dependent on the voltage unbalance factor (VUF). It also depends on the sequence-to-sequence angle, whose practical value is doubtful. Besides, the significant oscillations in the active power in the technique is a matter of concern. To reduce the complexity, the authors in^33^ proposed a current limitation strategy, which is independent of VUF. In^34^, the authors proposed an LVRT control technique that ensures a full harnessing of the rated capacity of distributed PV system during voltage sags. However, the strategy doesn’t work with the grid code compliances.

Considering the above-mentioned drawbacks, this paper presents a simple LVRT control technique that ensures that the power capabilities of distributed PV systems are fully utilized during voltage sags. To ensure smooth ride-through operation, a control strategy is formulated by considering three power generation scenarios. The proposed control strategy suggests certain reference currents along with positive and negative active and reactive power injections with characteristics, which are flexible and which can be adapted to simultaneously achieve the following objectives during voltage sags:

1. Injecting the maximum value of the rated current regardless of the profile of sag;
2. Providing current limitation to prevent activation of over current protection;
3. Exploiting fully the PV inverter’s maximum capacity;
4. Avoiding second order harmonic oscillations in real power and dc-link capacitor voltage under unbalanced sags.

The rest of the paper is organized as follows. “Problem characterization” section describes the challenges during power imbalance at dc-link and the importance of the overcurrent protection of the inverter under voltage sags. “Conventional strategies for generation of current reference” section briefly discusses the performance of major conventional strategies used in generation of reference current when the grid is witnessing an unbalanced voltage. “Proposed control technique” section provides the mathematical formulation of the proposed strategy during the LVRT period. “Results and discussion” section compares the performance of the proposed strategy with two state-of-the-art control strategies for different generation scenarios under balanced and unbalanced grid faults. Finally, the concluding remarks are presented to outline the benefits of the proposed strategy in “Conclusion” section.

## Problem characterization

During LVRT operation, an effective dc-link voltage control loop must be designed^4^. Normally, the power harnessed from PV plant is transferred to the grid via dc-link capacitor to guarantee power balance, under stable operating conditions on the grid. When a fault occurs in the grid, the voltage tends to dip requiring the LVRT feature to be activated following the grid codes to evaluate the reference of reactive power. The reference of the active power is set by the power rating of the inverter. In absence of any sag (${P}^{*}\ge {P}_{ac})$, the injected active power, (${P}_{ac}$) should adhere to the reference value (${P}^{*}$). When these values of power are not equal an imbalance in the system occurs.

### Power Imbalance at dc-link

**Figure 1:**
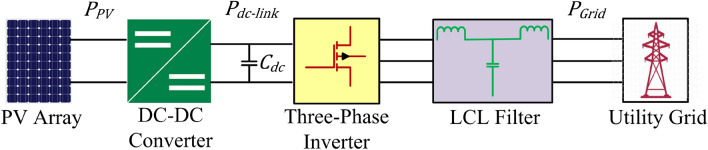
Block diagram of a two-stage grid-connected PV system.

As is evident from Fig. 1, in the case of a two-stage, 3-phase grid connected system, the total power from PV array, given by (1), is the sum of the power consumed in dc-link plus the power delivered to the grid.

1 $${P}_{PV} = {P}_{dc}{ + P}_{Grid}$$

where ${P}_{PV}$ is the PV system’s output power, ${P}_{dc}$, the power flowing within dc-link capacitor, and ${P}_{Grid}$ is the inverter’s power injection to the grid.

Under normal operating grid conditions,

2 $${P}_{PV}={P}_{Grid}=3{U}_{Grid}{I}_{Grid}$$

where ${U}_{Grid}$ and ${I}_{Grid}$ are the respective RMS values of the phase voltage and phase current. As can be seen from (2), if the losses in power converter are ignored, the voltage of the dc-link will be preserved under normal operating conditions.

However, under unbalanced voltage sag ${P}_{Grid}$ reduces to ${P}_{Grid\left(f\right)}$, where ${P}_{Grid\left(f\right)}$ is the power injected by the inverter post fault. Meanwhile, the dc-dc converter continues to inject the dc-link with all the available power from PV system. The voltage at the dc-link will rise significantly as a result of this imbalance between ${\text{P}}_{\text{PV}}$ and ${P}_{Grid\left(new\right)}$. The mathematical representation of the power difference condition is given in (3).

3 $$\left({P}_{PV}-{P}_{Grid\left(f\right)}\right)\Delta t={P}_{dc}\Delta t=\frac{1}{2}{C}_{dc}\left({U}_{dc,f}^{2}-{U}_{dc}^{2}\right)$$

Here ${U}_{dc}$ and ${U}_{dc,f}$ are the dc-link voltages before and after fault, respectively. $\Delta t$ shows the time duration of fault. Considering (2) and (3) and taking ${P}_{Grid\left(f\right)}=3{U}_{dc,f}{I}_{Grid}$, the voltage at dc-link during fault is obtained, as in (4).

4 $${U}_{dc,f} =\sqrt{\frac{2\left({P}_{PV}-3{U}_{dc,f}{I}_{Grid}\right)\Delta t}{{C}_{dc}}+{U}_{dc}^{2}}$$

It is evident from (4) that the dc-link voltage will rise during fault with deeper voltage sag and longer time duration of the fault. Hence, protective schemes are imperative to avoid overvoltage violation at the dc-link. In order to avoid the activation of over voltage protection device, which may disconnect the inverter from the grid, the PV should stop operating at its MPP and the PV power must be curtailed down to a safe value.

### Inverter over current protection

Furthermore, under unbalanced grid voltage conditions, the inverter should inject reactive power to provide voltage support at PCC, the point of common coupling. Hence, the inverter is used to inject reactive power in an appropriate amount. The grid code prescribes this amount, based on as to how severe is the dip in the grid voltage. As the power system operators require injection of reactive power from PVs during period of low-voltage-ride-through. The situation is complicated further if the PV keeps operating at its MPP. The simultaneous injection of peak active power from the PV array, as well as the requirement of injecting the reactive power by the inverter can cause an over current in the inverter. Due to this over current, the inverter protection system can turn on and disconnect the inverter as a protective measure during low-voltage-ride-through period. As a result, current limiting is a key goal in LVRT to restrict the amplitude of injected currents to a value within the rated limits of the inverter in order to obviate the chance of triggering the over-current relay.

## Conventional strategies for generation of current reference

**Figure 2:**
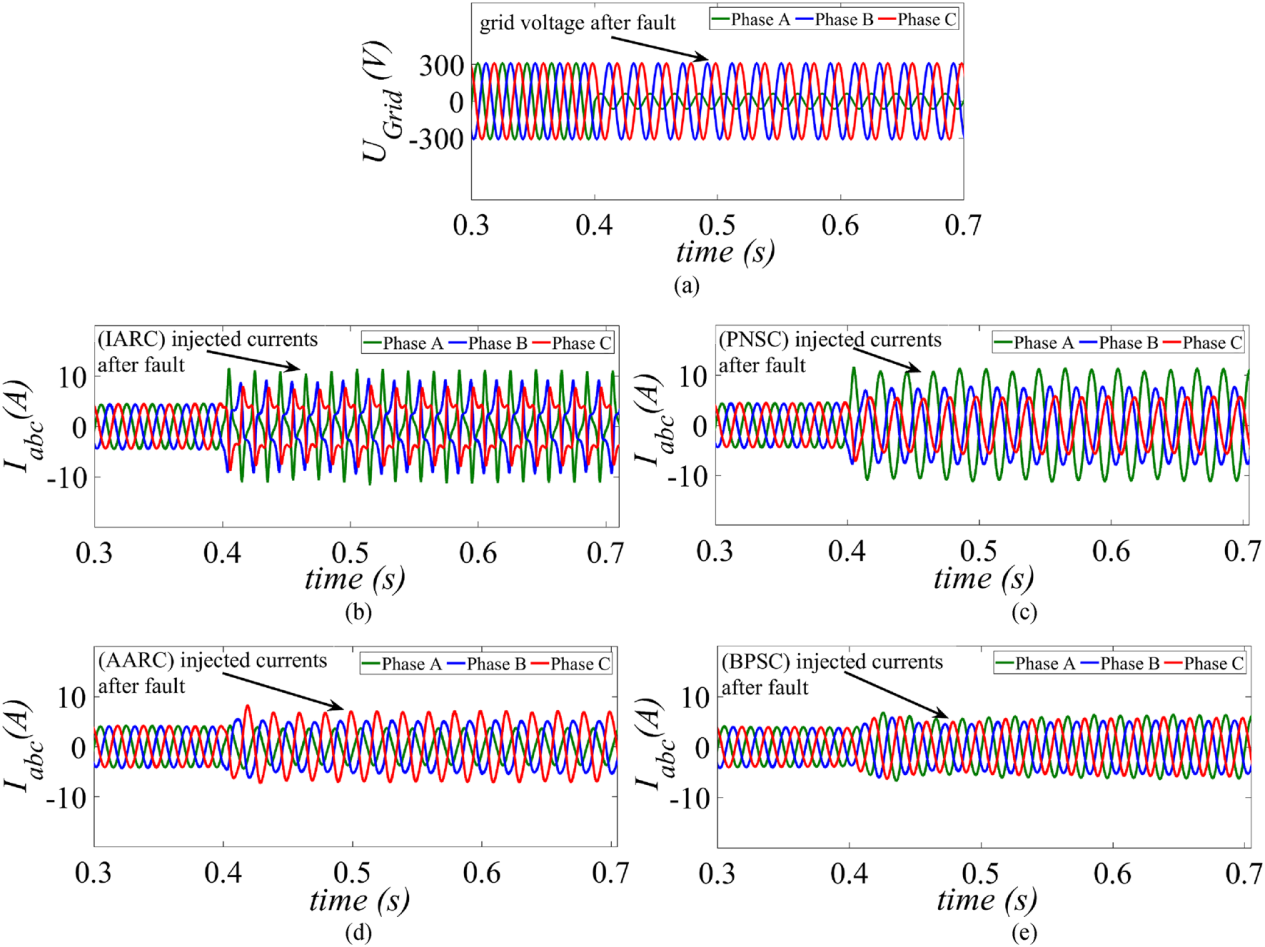
Conventional current control strategies (**a**) Grid voltage during a single-phase dip (**b**) Instantaneous active reactive control (IARC) (**c**) Positive negative sequence control (PNSC) (**d**) Average active reactive control (AARC) (**e**) Balanced positive sequence control.

As mentioned in “Introduction” section, for injecting active and reactive power to the grid, a reference current corresponding to the demand is required to be generated, and a proper strategy for generating this current reference (CRG) in accordance with the applicable grid code is essential. A review of four existing reference generation strategies IARC, AARC, PNSC, BPSC for three-phase GC PV systems follows. The simulation results of these conventional CRG techniques are also verified using MATLAB/Simulink. To illustrate the behaviour of conventional CRG techniques a voltage dip is created at t = 0.4 s as shown in Fig. 2a.

### Instantaneous active reactive control (IARC)

In concept, the IARC based control scheme helps a grid under UPF to get the instantaneous active and reactive power quite effectively. The required reference currents for active and reactive power are computed using (5).

5 $$i^{*} = i_{p}^{*} + i_{q }^{*} = \frac{1}{{\left| {u^{2} } \right|}}\left[ {\begin{array}{*{20}c} P & Q \\ \end{array} } \right]\left[ {\begin{array}{*{20}c} u \\ { u_{ \bot } } \\ \end{array} } \right]$$

where ${\text{P}}$ and ${\text{Q}}$, in turn, indicate the set points for the active and reactive power. As mentioned earlier, this scheme injects sinusoidal currents under unity power factor only under balanced grid conditions. The scheme does not inject reactive power due to the absence of orthogonal component of voltage vector ($u_{ \bot }$). During unbalanced grid conditions, the injected currents exhibit a heavy distortion because of the presence of higher order harmonics caused by double frequency oscillations as given in (6).

6 $${\left|u\right|}^{2}={\left|{u}^{+}\right|}^{2}+{\left|{u}^{-}\right|}^{2}+2\left|{u}^{+}\right|\left|{u}^{-}\right|cos\left(2\omega t+{\varnothing }^{+}-{\varnothing }^{-}\right)$$

where $\varnothing^{ + }$ is the phase angle of the positive sequence voltage vector ${u}^{+},$ and $\varnothing^{ - }$ the phase angles of negative sequence voltage vector, ${u}^{-}$.

### Positive–negative sequence control (PNSC)

This strategy controls the positive sequence and the negative sequence components in the reference equations in an effective manner, and thereby eliminates second harmonic oscillations. Certain oscillation effects can be cancelled out during unbalanced faults in three-phase GCPV systems by using the PNSC method. As shown in Fig. 2b, using PNSC strategy sinusoidal, but unbalanced grid currents are obtained. The set of reference currents for active and reactive power are calculated as in (7).

However, the interplay between the positive sequence component and negative sequence component results in large swinging of reactive power. From this standpoint, it appears that the PNSC method is not a very attractive choice for three-phase PV inverters during unsymmetrical faults in the grid.

7 $${i}^{*}={{i}_{p}}^{*}{+{i}_{q}}^{*}=\frac{1}{\left[{\left|{u}^{+}\right|}^{2}+{\left|{u}^{-}\right|}^{2}\right]}\left[\begin{array}{cc}{p}_{ref}& {q}_{ref}\end{array}\right]\left[\begin{array}{c}{u}^{+}-{u}^{-}\\ {u}_{\perp }^{+}-{u}_{\perp }^{-}\end{array}\right]$$

### Average active reactive control (AARC)

While discussing IARC it was mentioned that harmonics are injected into the grid during unbalanced grid condition as the instantaneous conductance ($g$) and susceptance ($b$) are not constant throughout one grid period ($T$). It was already assumed that ${\text{P}}$ and ${\text{Q}}$ are constant, therefore these harmonics in the reference current vector, occur due to the presence of second order component of $\left|{u}^{2}\right|$ as in (6). To alleviate the effect of these $\left|{u}^{2}\right|$ oscillations from $g$ and $b$, their average value is calculated.

As depicted from Fig. 2c, the behaviour of injected currents in AARC is similar to PNSC. Moreover, there are no oscillations in instantaneous reactive power whereas, oscillations at twice the fundamental frequency of grid, are observed in the instantaneous active power. Hence, the reference current vectors ${i}_{p}^{*}$ and ${i}_{q }^{*}$ are now obtained as in (8):

8 $$\left[\begin{array}{c}{i}_{p}^{*}\\ {i}_{q}^{*}\end{array}\right]=\frac{P}{\left[{{u}_{\alpha }}^{+2}+{{u}_{\beta }}^{+2}\right]-\left[{{u}_{\alpha }}^{-2}+{{u}_{\beta }}^{-2}\right]}\left[\begin{array}{c}{u}_{\alpha }^{+}+{u}_{\alpha }^{-}\\ {u}_{\beta }^{+}+{u}_{\beta }^{-}\end{array}\right]$$

### Balanced positive sequence control (BPSC)

When the injected reference currents are required to be free from harmonics, BPSC strategy is used under LVRT^13^. In this strategy, balanced and sinusoidal currents are supplied to the grid. The scheme injects only positive sequence component and is based on the same principle as AARC.

It can be clearly seen from Fig. 2d, this control method produces balanced and sinusoidal output currents during LVRT operation. Active and reactive current reference in this scheme is obtained as:

9 $$\left[\begin{array}{c}{i}_{\alpha p}^{*}\\ {i}_{\alpha q}^{*}\end{array}\right]=\frac{P}{{{u}_{\alpha }}^{+2}+{{u}_{\beta }}^{+2}}\left[\begin{array}{c}{u}_{\alpha }^{+}\\ {u}_{\beta }^{+}\end{array}\right]$$

As previously mentioned, the conventional CRG strategies require modifications so that during unbalanced fault an uninterrupted feed to the grid from DG is maintained^18^. This modification becomes essential as the conventional strategies are not able to help in voltage support or be able to limit the high currents, the basic requirements of LVRT^19^. In absence of any current limiting feature in conventional strategies, high peak currents can be noticed in Fig. 2 for all the four strategies just reviewed. These uncontrolled high peak values of current may activate the protective relays and cut the DG resources off the grid.

To overcome the drawbacks in the above mentioned CRG strategies, a new improved strategy, proposed by authors, is discussed in the following section.

## Proposed control technique

**Figure 3:**
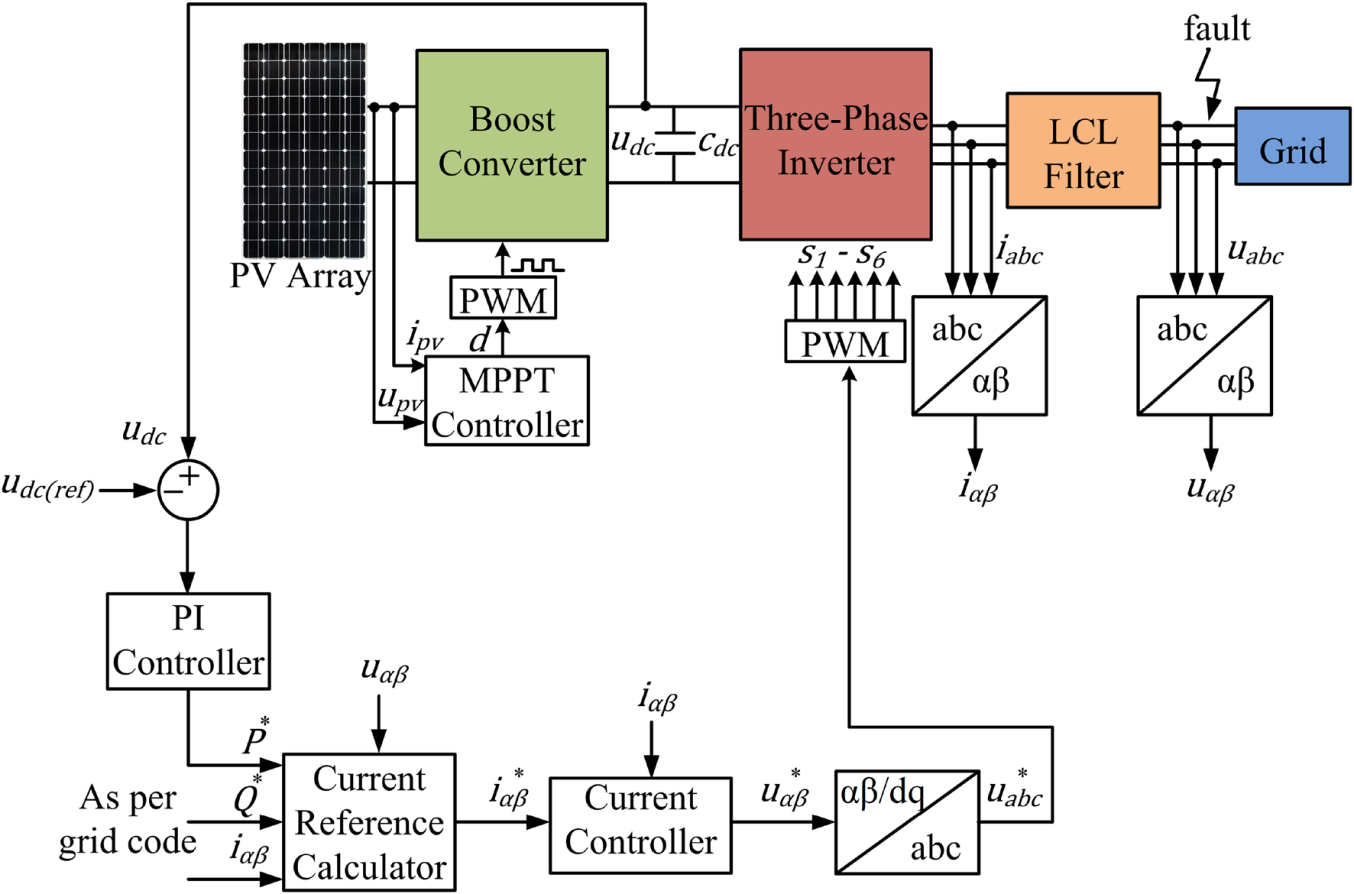
Block diagram of the proposed control strategy.

Figure 3 shows the complete scheme of the proposed control strategy for a three-phase three-wire two stage grid connected PV system. The three-phase grid voltages are converted into the stationary reference frame (SRF) as in (10).

10 $${u}_{\alpha \beta }=\left[\begin{array}{c}{u}_{\alpha }\\ {u}_{\beta }\end{array}\right]=\sqrt{\frac{2}{3}} \left[\begin{array}{ccc}1& -1/2& -1/2\\ 0& \sqrt{3/2}& -\sqrt{3/2}\end{array}\right]\left[\begin{array}{c}{u}_{a}\\ {u}_{b}\\ {u}_{c}\end{array}\right]$$

where ${u}_{\alpha }$, ${u}_{\beta }$ are the voltages in the SRF and ${\text{u}}_{\text{a}}$, ${\text{u}}_{\text{b}}$, ${\text{u}}_{\text{c}}$ are the grid voltages in the a-b-c frame of reference, respectively. Instantaneous values of active and reactive power of a three-phase GCPV inverter are given in (11) and (12), respectively.

11 $$\mathrm{p }=\mathrm{ u}\cdot{\text{i}}$$

12 $$\mathrm{q }= {{\text{u}}}_{\perp }\cdot\mathrm{i }=\mathrm{ u}\times {\text{i}}$$

Further, the apparent power *S* is written as in (13)

13 $$S= u\cdot{i}^{*}=P+jQ$$

where ${\text{p}}$ and ${\text{q}}$ respectively represents the active and reactive power delivered by the inverter, $\mathrm{u }={\mathrm{ u}}_{{\text{abc}}}$ is the voltage vector and ${\text{i}}={i}_{abc}$ is the injected current vector at the PCC. Whereas ${u}_{\perp }$ is the orthogonal component of the grid voltage vector. Unlike in balanced grid conditions, the positive and negative sequence components of voltage and currents appear during unbalanced voltage conditions.

The voltage, current and their orthogonal component of voltage ${u}_{\perp }$, under unbalanced grid conditions are given by (14), (15) and (16), respectively. Each expression contains two terms, the positive sequence component and the negative one.

14 $$u = {u}^{+}+ {u}^{-}$$

15 $$i = {i}^{+}+{ i}^{-}$$

16 $${u}_{\perp }= {{u}_{\perp }}^{+}+{{u}_{\perp }}^{-}$$

where ${u}^{+}$, ${u}^{-}$ and ${i}^{+}$, ${i}^{-}$ are the positive and negative sequence component of voltage and current, respectively. Since the system is a three-phase three-wire, zero sequence components are not considered. The apparent power $S$ is now rewritten as in (17).

17 $$S = u\cdot{i}^{*} = P+jQ$$

where $u$ and $i$ are the voltage and current vectors in the SRF, and $P$, $Q$ are the active and reactive power, respectively. Under normal operating conditions, the grid voltages are balanced and hence, negative sequence component and oscillatory components are absent in the injected active and reactive power. On the other hand, the voltages and currents contain negative sequence components under unbalanced grid voltage conditions. Hence (17) can be re-written as:

18 $${S=u}_{\alpha \beta }\cdot{{i}_{\alpha \beta }}^{*}=\left({u}_{\alpha \beta }^{+}+{u}_{\alpha \beta }^{-}\right)\cdot{\left({i}_{\alpha \beta }^{+}+{i}_{\alpha \beta }^{-}\right)}^{*}= {u}_{\alpha \beta }^{+}.{{i}_{\alpha \beta }^{+}}^{*}+{u}_{\alpha \beta }^{+}{\cdot{i}_{\alpha \beta }^{-}}^{*}+{u}_{\alpha \beta }^{-}\cdot{{i}_{\alpha \beta }^{+}}^{*}+{u}_{\alpha \beta }^{-}\cdot{{i}_{\alpha \beta }^{-}}^{*}$$

where

19 $${u}_{\alpha \beta }^{+}=\frac{1}{2}\left[\begin{array}{cc}1& -{e}^{-j\pi /2} \\ {e}^{-j\pi /2} & 1\end{array}\right]{u}_{\alpha \beta } and {u}_{\alpha \beta }^{-}=\frac{1}{2}\left[\begin{array}{cc}1& {e}^{-j\pi /2} \\ -{e}^{-j\pi /2} & 1\end{array}\right]{u}_{\alpha \beta }$$

where ${e}^{-j\pi /2}$ is a 90° degree lagging phase-shifting operator. Hence, (19) can be expanded as below:

20 $${u}_{\alpha \beta }^{+}\cdot{{i}_{\alpha \beta }^{+}}^{*}={(u}_{\alpha }^{+}+j{u}_{\beta }^{+}).{{(i}_{\alpha }^{+}+j{i}_{\beta }^{+})}^{*}{=u}_{\alpha }^{+}{i}_{\alpha }^{+}+{u}_{\beta }^{+}{i}_{\beta }^{+}+j\left({u}_{\beta }^{+}{i}_{\alpha }^{+}-{u}_{\alpha }^{+}{i}_{\beta }^{+}\right)$$

From (20), the constant and oscillating terms in active and reactive power respectively can be obtained.

In the proposed control strategy, the current references are formulated in stationary reference frame by eliminating the double-grid frequency oscillations from the injected active power and dc-link voltage. The mathematical expression formulated for the reference currents for active and reactive power are given in (21–24).

21 $${i}_{\alpha A} = \frac{{u}_{\alpha }^{+}-{u}_{\alpha }^{-}}{\left({u}_{\alpha }^{{+}^{2}}+{u}_{\beta }^{{+}^{2}}\right)+{ X}_{\alpha A }\left({u}_{\alpha }^{{-}^{2}}+{u}_{\beta }^{{-}^{2}}\right)}{P}^{*}$$

22 $${i}_{\beta A} =\frac{{u}_{\beta }^{+}-{u}_{\beta }^{-}}{\left({u}_{\alpha }^{{+}^{2}}+{u}_{\beta }^{{+}^{2}}\right)+{ X}_{\beta A}\left({u}_{\alpha }^{{-}^{2}}+{u}_{\beta }^{{-}^{2}}\right)}{P}^{*}$$

23 $${i}_{\alpha R} =-\frac{{u}_{\alpha \perp }^{+}+{u}_{\alpha \perp }^{-}}{\left({u}_{\alpha \perp }^{{+}^{2}}+{u}_{\beta \perp }^{{+}^{2}}\right)+{ X}_{\alpha R }\left({u}_{\alpha \perp }^{{-}^{2}}+{u}_{\beta \perp }^{{-}^{2}}\right)}{Q}^{*}$$

24 $${i}_{\beta R} =-\frac{{u}_{\beta \perp }^{+}+{u}_{\beta \perp }^{-}}{\left({u}_{\alpha \perp }^{{+}^{2}}+{u}_{\beta \perp }^{{+}^{2}}\right)+{ X}_{\beta R}\left({u}_{\alpha \perp }^{{-}^{2}}+{u}_{\beta \perp }^{{-}^{2}}\right)}{Q}^{*}$$

where ${i}_{\alpha A}$, ${i}_{\beta A}$, ${i}_{\alpha R}$ and ${i}_{\beta R}$ are the active and reactive current references in the stationary reference frame. It is important to point that the denominator factors ${X}_{\alpha A}$, ${X}_{\beta A}$, ${X}_{\alpha R}$ and ${X}_{\beta R}$ are taken as -1 to exploit the inverter’s maximum capacity.

To provide overcurrent limitation as well as to ensure maximum exploitation of the inverter capacity the performance of the proposed control strategy, is evaluated as per the three generation scenarios given below:

### High power generation scenario (Irradiance ranging from 1000 W/m^2^–800 W/m^2^)

In this case, the inverter’s capacity is majorly exploited through the injection of active power under normal operating condition. To provide voltage support at the PCC, reactive power is injected into the grid under fault conditions as per the specified grid codes. As previously discussed, the simultaneous injection of peak active power from PVs and reactive power into the grid for voltage support can trigger the over current protection mechanism in PV inverter. The triggering of over current protection will lead to disconnection of inverter from the grid which is unfavourable during LVRT period. As the injection of reactive power is mandatory as per the grid code, only the remaining capacity of the inverter can be used to inject active power from PV array. Hence, to avoid over current in PV inverters during fault-ride-through period, active power curtailment is necessary. The authors have formulated an expression to evaluate pseudo inverter capacity (PIC) for over current limitation as in (25).

25 $$PIC= \frac{1-VUF}{{u}_{base}}\times {u}^{+}\times S$$

where the voltage unbalance factor (VUF) ranges between 0–1 and is defined as in (26).

26 $$VUF= \frac{{u}^{-}}{{u}^{+}}$$

Based on the voltage sag depth the reactive power reference is obtained as given in (27)^35^.

27 $$\left\{ {\begin{array}{*{20}l} {Q^{*} = 0} \hfill & {if\;u_{pu} > 0.9} \hfill \\ {Q^{*} = S \times 1.5 \times \left( {0.9 - U_{pu} } \right)} \hfill & {if\;0.2 < U_{pu} < 0.9} \hfill \\ {Q^{*} = 1.05 \times S} \hfill & {if\;U_{pu} < 0.2} \hfill \\ \end{array} } \right.$$

where $${u}_{pu}=\frac{\sqrt{{u}_{\alpha }^{2}+{u}_{\beta }^{2}}}{u}$$

The maximum value of active power (${P}_{max}$) that can be injected into the grid without triggering the overcurrent protection is given in (28).

28 $${P}_{max}= \sqrt{{PIC}^{2}-{Q}^{2}}$$

In case of severe grid voltage dip, the value of PIC will be very less because ($1-{\text{VUF}}$) will be small, and under those conditions $\left( {Q > PIC} \right) \to = PIC, \;{\text{and}}\;P_{\max } = 0$.

This indicates that the converter cannot inject that much reactive power to the grid if the reactive power reference is higher than the PIC. As a result, the PIC is chosen as reactive power reference, and no power is extracted from the PV arrays.

**Figure 4:**
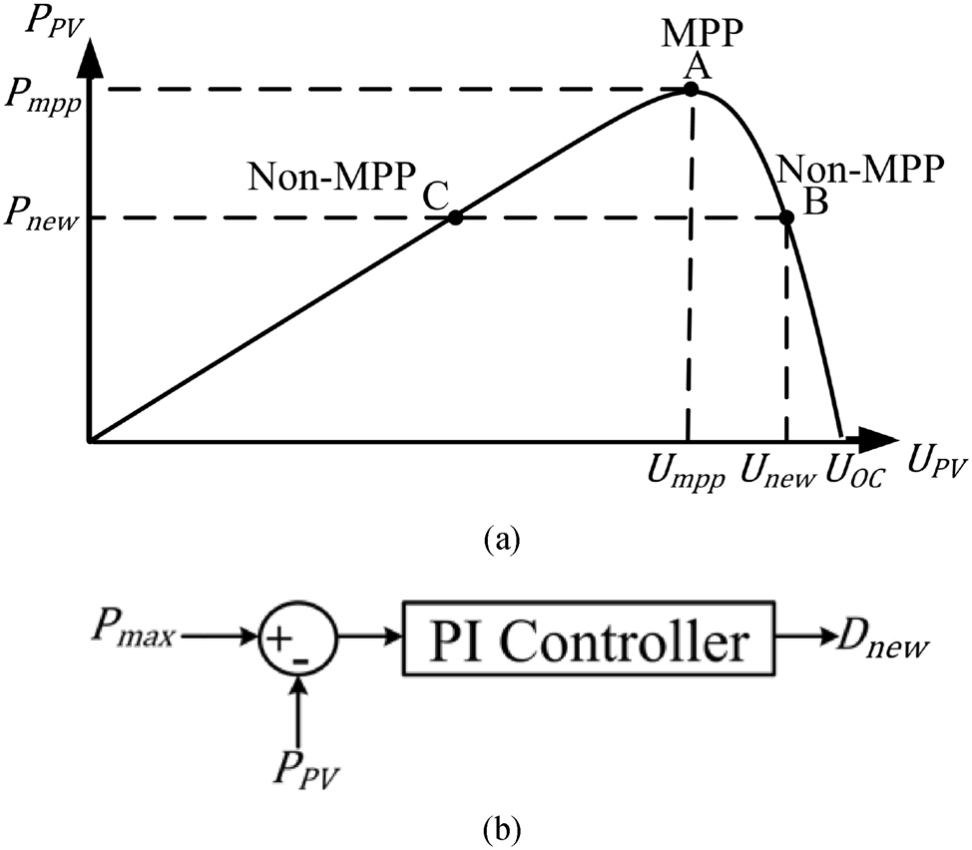
Non-MPPT mode of operation: (**a**) point of operation on power curve and (**b**) control block.

Under voltage sag condition, ${P}_{max}$ is constantly compared with ${P}^{*}$ and if ${P}_{max}>{P}^{*}$, the inverter keeps delivering the same amount of active power. The P&O algorithm for MPPT is terminated as soon as ${P}_{max}< {P}^{*}$, is reached, and to prevent over voltage in the dc-link capacitor, the point of operation is relocated to non-MPP point ${P}_{max}$ as shown in Fig. 4a.

The duty cycle for the non-MPPT mode is obtained as in Eq. (29). The control block diagram to determine the duty cycle for the non-MPPT mode of operation is shown in Fig. 4b. The non-MPPT mode is operated on the right side of the PV characteristics as the non-MPPT point is close to the MPP.

29 $${D}_{max} ={ K}_{p} ({P}_{max}-{P}_{PV}) + \frac{{K}_{i}}{s} ({P}_{max}-{P}_{PV})$$

The non-MPPT mode of operation is carried out to reduce active power from PV array which limits over current in the PV inverter. In this case, the active power is practically free of oscillation, but the injected reactive power oscillates at twice the grid frequency.

### Medium power generation scenario (Irradiance ranging from 700 W/m^2^–400 W/m^2^)

In this case, the inverter capacity is exploited by partially injecting both active and reactive power under fault conditions. Since the generated active power is not high, the remaining inverter capacity is utilized by injecting reactive power as in (30).

30 $${Q}_{\left({G}_{700-}{G}_{400}\right)}=\sqrt{{S}^{2}-{{P}^{2}}_{\left({G}_{700-}{G}_{400}\right)}}$$

### Low power generation scenario (Irradiance ranging from 300 W/m^2^–100 W/m^2^)

Since, the active power generated is very low, no active power curtailment is required and hence, over current protection is not activated. In this case, the inverter capacity is majorly exploited by injecting reactive power as in (31).

31 $${Q}_{\left({G}_{300-}{G}_{100}\right)}=\sqrt{{S}^{2}-{{P}^{2}}_{\left({G}_{300-}{G}_{100}\right)}}$$

## Results and discussion

To demonstrate the effectiveness of the proposed current control strategy, three case studies have been carried out in MATLAB/Simulink environment. The parameters of GCPV system are given in Table 1. Moreover, the proposed control strategy is compared with two prior-art control strategies^32,33^.

**Table 1:** Parameters for the proposed GCPV system.

Software	PV (W)	Grid Line-Line Voltage	dc-link capacitor	dc-link voltage	L_in_	L_g_	C_f_	f_sw_
MATLAB/Simulink	2 kW	381 V, 50 Hz	1360 µF	697 V	0.05 H	0.025 H	20 µF	2500 Hz

This section entails the results of a 2-kW grid connected PV system under unbalanced grid voltage sag conditions using the parameters shown in Table 1.

### Transformer Less VSS (TL-VSS)^32^ vs proposed strategy during single-phase voltage sag under high power generation scenario

**Figure 5:**
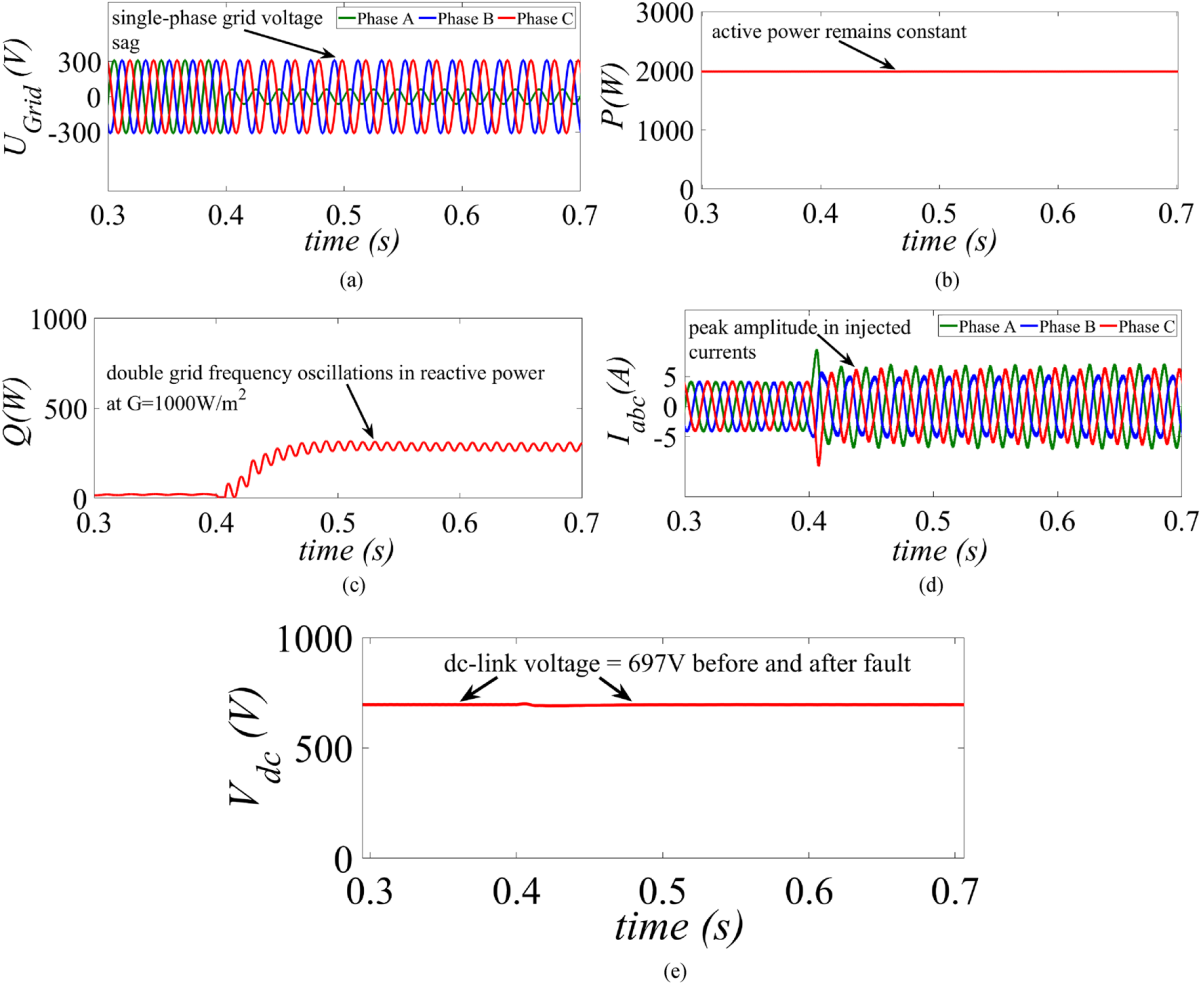
Results of the TL-VSS under high power generation scenario at G = 1000 W/m^2^ (**a**) grid voltage, (**b**) injected active power, (**c**) injected reactive power, (**d**) inverter current and (**e**) dc-link voltage.

In^36^, the authors proposed a transformer less VSS (TL-VSS) which is used to compare the performance of the proposed control strategy. The comparison is carried out for high power generation scenario where the PV is operated at G = 1000 W/m^2^. In order to analyse the behaviour of the control strategies, an unbalanced fault is created at t = 0.4 s which triggers the low-voltage-ride-through period. The unbalanced fault is created by reducing the phase A voltage from 1 to 0.2 p.u whereas phase B and C remain unchanged as shown in Fig. 5a. The reference value of the dc-link voltage is taken as $\text{1.3}\sqrt{2}{{\text{U}}}_{\text{L-L, RMS}}$ which is equal to 697 V.

The maximum power extracted from the PV array is 2 kW before and after fault under STC at G = 1000 W/m^2^ using the TL-VSS is shown in Fig. 5b. Since, the TL-VSS does not provide current limitation the injected currents are above the rated capacity of the inverter (5A) as shown in Fig. 5d. The reactive power remains zero before fault and starts increasing after the fault as per the specified grid code in (22). It is evident that the injected reactive power has double-grid frequency oscillations as shown in Fig. 5c. Although, TL-VSS provides good voltage support, it fails to provide safe operation of the inverter during low-voltage-ride-through period and can lead to disconnection of the PV inverter under high power generation scenarios.

On the other hand, the proposed current limitation control strategy is formulated to ensure injected currents do not surpass the rated capacity of the inverter as well as maximum exploitation of the inverter capacity is guaranteed.

**Figure 6:**
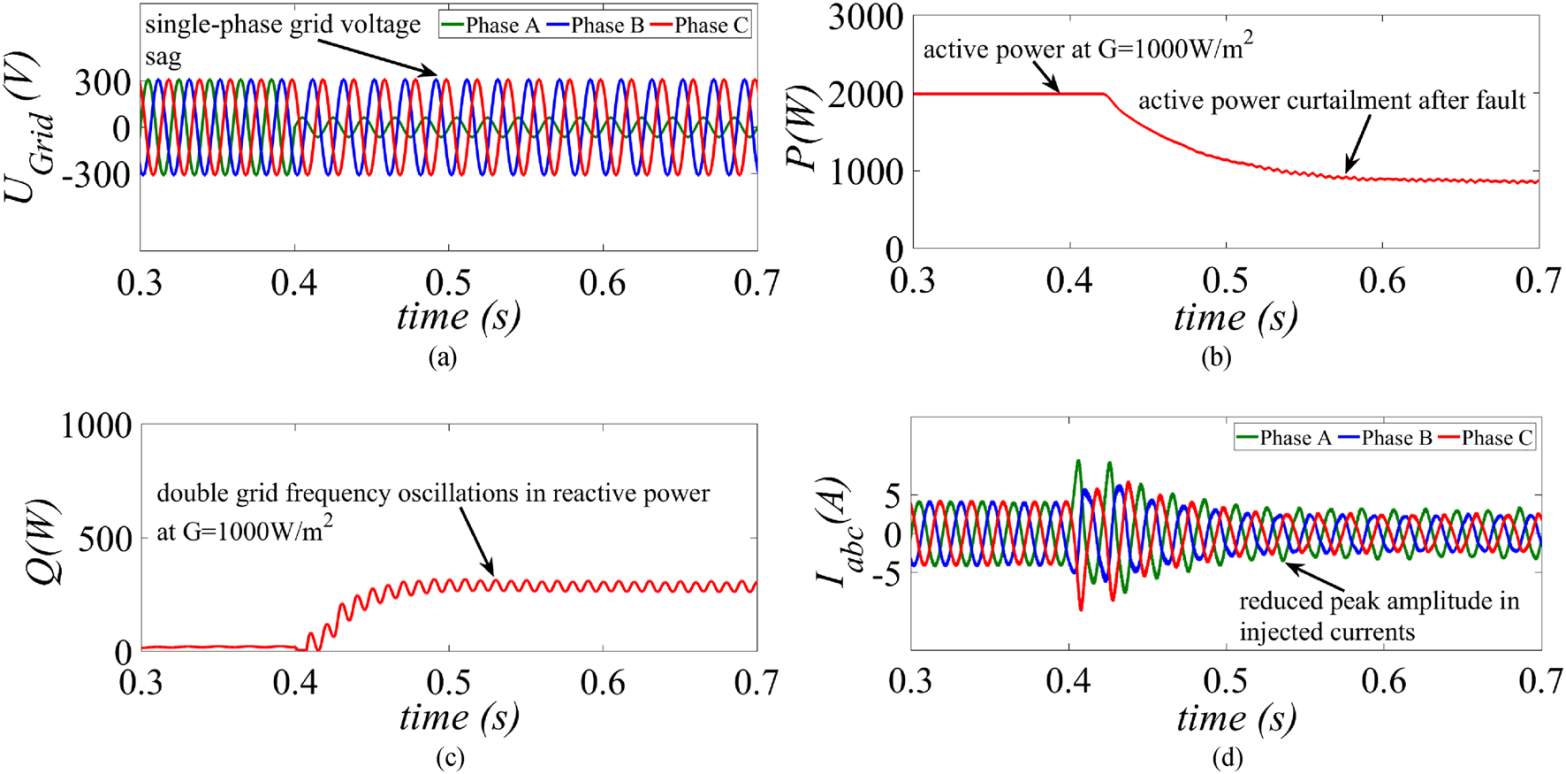
Results of the proposed control strategy under high power generation scenario at G = 1000 W/m^2^ (**a**) grid voltage, (**b**) injected active power, (**c**) injected reactive power and (**d**) inverter current.

The proposed current limitation control strategy is tested on a similar fault condition where the phase A voltage is reduced to 0.2 p.u. at t = 0.4 s as shown in Fig. 6a. It was observed in Fig. 6b, the mean value of the active power is 2 kW before the fault. During low-voltage-ride-through period reactive power must be injected according to the grid code as given in (27). The injected reactive power in this case is 300 VAr as per the specified grid code. Hence, during fault period, the GCPV inverter must be protected from over current by activating the APC control loop which limits the active power to ${P}_{max}$ and the new reference power ${P}^{*}={P}_{max}$ which is measured around 920 W. Therefore, to exploit the maximum capacity of the inverter both reactive and active power is injected into the grid without triggering over current protection. The reactive power is allowed to oscillate at double grid frequency as shown in Fig. 6c. It is evident from Fig. 6d, that the peak amplitude of the injected currents is reduced, and the control loop also tries to balance the phase currents. At t = 0.4 s, when the fault occurs there is fluctuation in the dc-link voltage. However, it quickly settles back to its initial value of 697 V as shown in Fig. 6d. As compared to TL-VSS, the proposed current limitation strategy shows better dynamic performance and hence the peak amplitude of the injected current is reduced within one cycle.

### Margin Residue CLS (MR-CLS)^37^ vs proposed control strategy during single-phase voltage sag under medium and low power generation scenario

**Figure 7:**
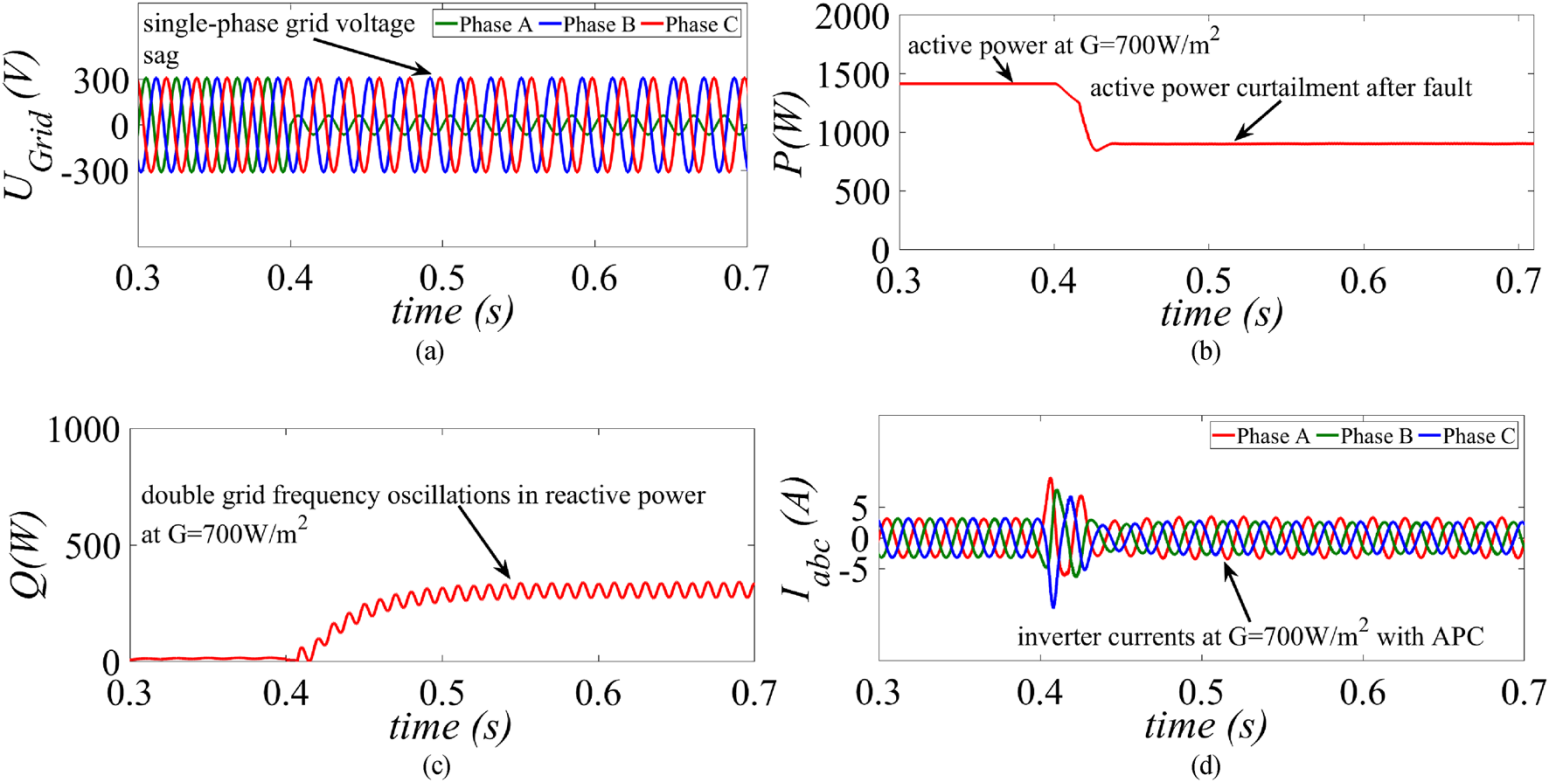
Results of the MR-CLS under medium power generation scenario at G = 700 W/m^2^ (**a**) grid voltage, (**b**) injected active power, (**c**) injected reactive power and (**d**) inverter current.

The performance of the proposed control strategy is compared with margin residue current limitation strategy (MR-CLS) of^33^ during single-phase grid voltage sag under medium (G = 700 W/m^2^) and low power (G = 300 W/m^2^) generation condition. A similar single-phase unbalanced fault is created at t = 0.4 s as shown in Fig. 7a. The average value for the active power before fault is around 1.4 kW as shown in Fig. 7b. As soon as the fault occurs, MR-CLS curtails down the active power to 920 W. At the same instant the reactive power starts increasing and reaches its steady-state value at 300 VAr as shown in Fig. 7c. Although the inverter currents are well below the rated capacity, the MR-CLS fails to exploit the maximum inverter capacity as shown in Fig. 7d.

**Figure 8:**
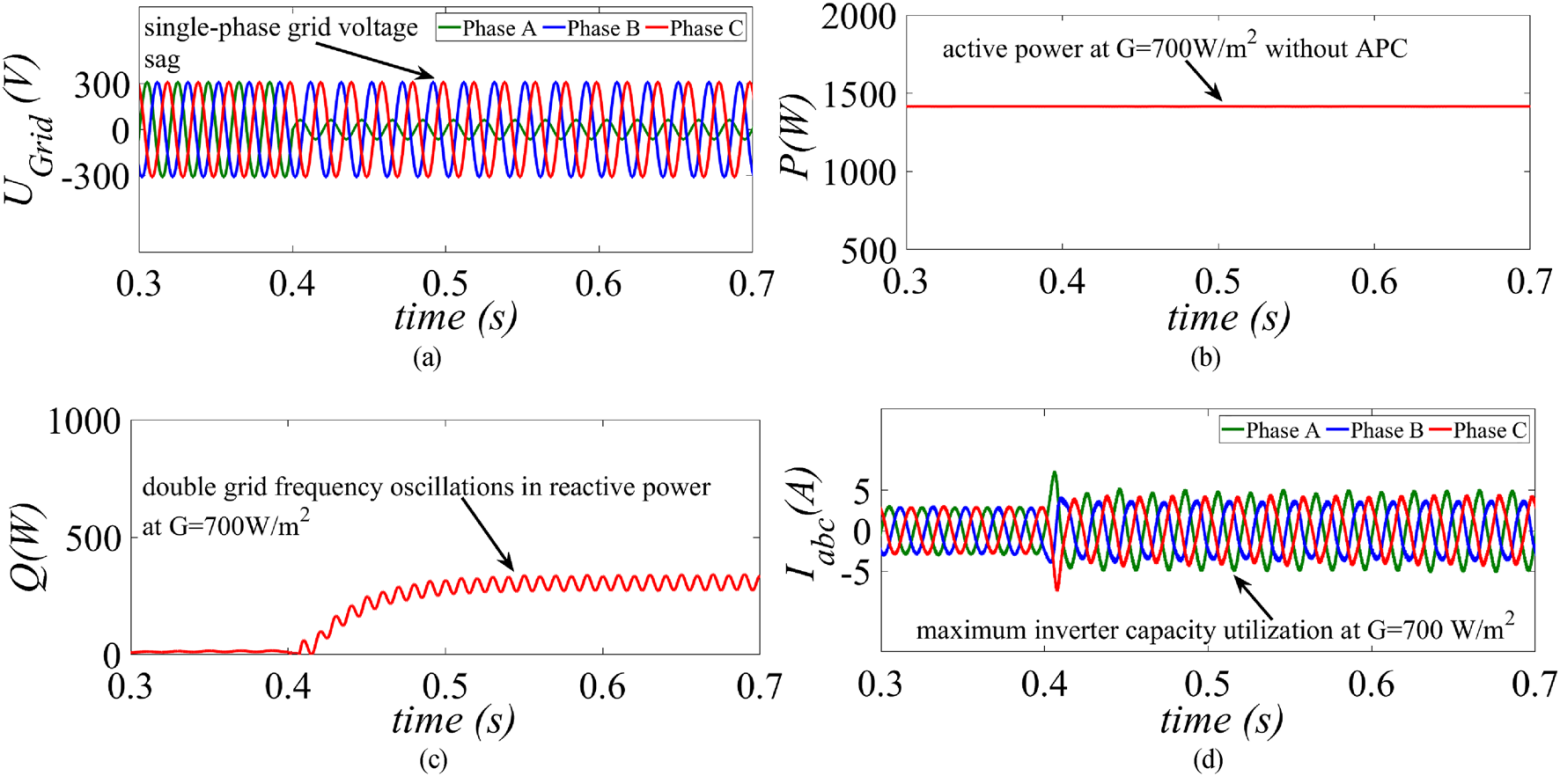
Results of the proposed control strategy under medium power generation scenario at G = 700 W/m^2^ (**a**) grid voltage, (**b**) injected active power, (**c**) injected reactive power and (**d**) inverter current.

On the other hand, under a single-phase unbalanced grid voltage sag as shown in Fig. 8a, the proposed control strategy injects maximum active power into the grid without surpassing the rated inverter capacity without activating the APC control loop as shown in Fig. 8b. The same amount of reactive power of 300 Var as per the specified grid code is injected into the grid as shown in Fig. 8c. It is clearly evident that maximum exploitation of the inverter’s capacity is achieved due to simultaneous injection of active and reactive power without curtailing the active power as shown in Fig. 8d.

**Figure 9:**
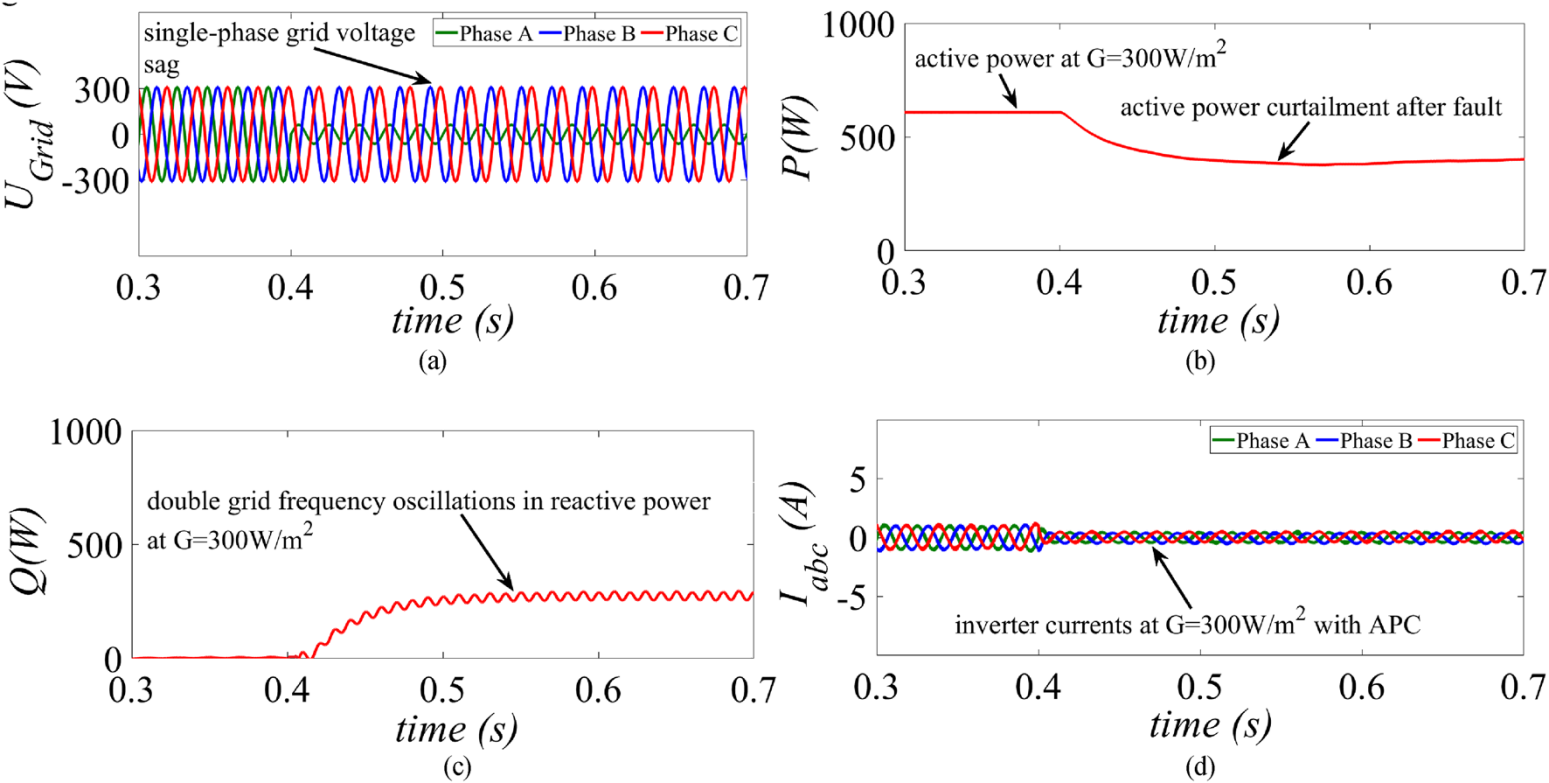
Results of the MR-CLS under low power generation scenario at G = 300 W/m^2^ (**a**) grid voltage, (**b**) injected active power, (**c**) injected reactive power and (**d**) inverter current.

Under low power generation condition, the GCPV system is operated at G = 300 W/m^2^ and at t = 0.4 s a single-phase unbalanced fault is created as shown in Fig. 9a. The MR-CLS reduces the active power around 390 W post fault as shown in Fig. 9b. The reactive power suffers from double grid frequency oscillations as shown in Fig. 9c. The inverter current amplitude is drastically reduced due to substantial curtailment in the active power and majority of the inverter capacity remain unutilized as shown in Fig. 9d.

**Figure 10:**
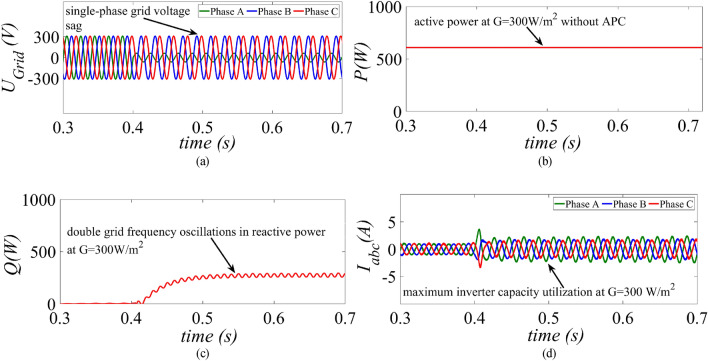
Results of the proposed control strategy under low power generation scenario at G = 300 W/m^2^ (**a**) grid voltage, (**b**) injected active power, (**c**) injected reactive power and (**d**) inverter current.

A single-phase unbalanced grid voltage sag is created as shown in Fig. 10a. During post fault period, the injected active power remains unchanged using the proposed strategy as shown in Fig. 10b. The reactive power magnitude remains the same as shown in Fig. 10c. Since, APC is not activated inverter’s capacity is exploited due to simultaneous injection of the available active and specified reactive power into the grid as shown in Fig. 10d.

### Performance evaluation of the proposed control strategy under two-phase and three-phase grid voltage sag

**Figure 11:**
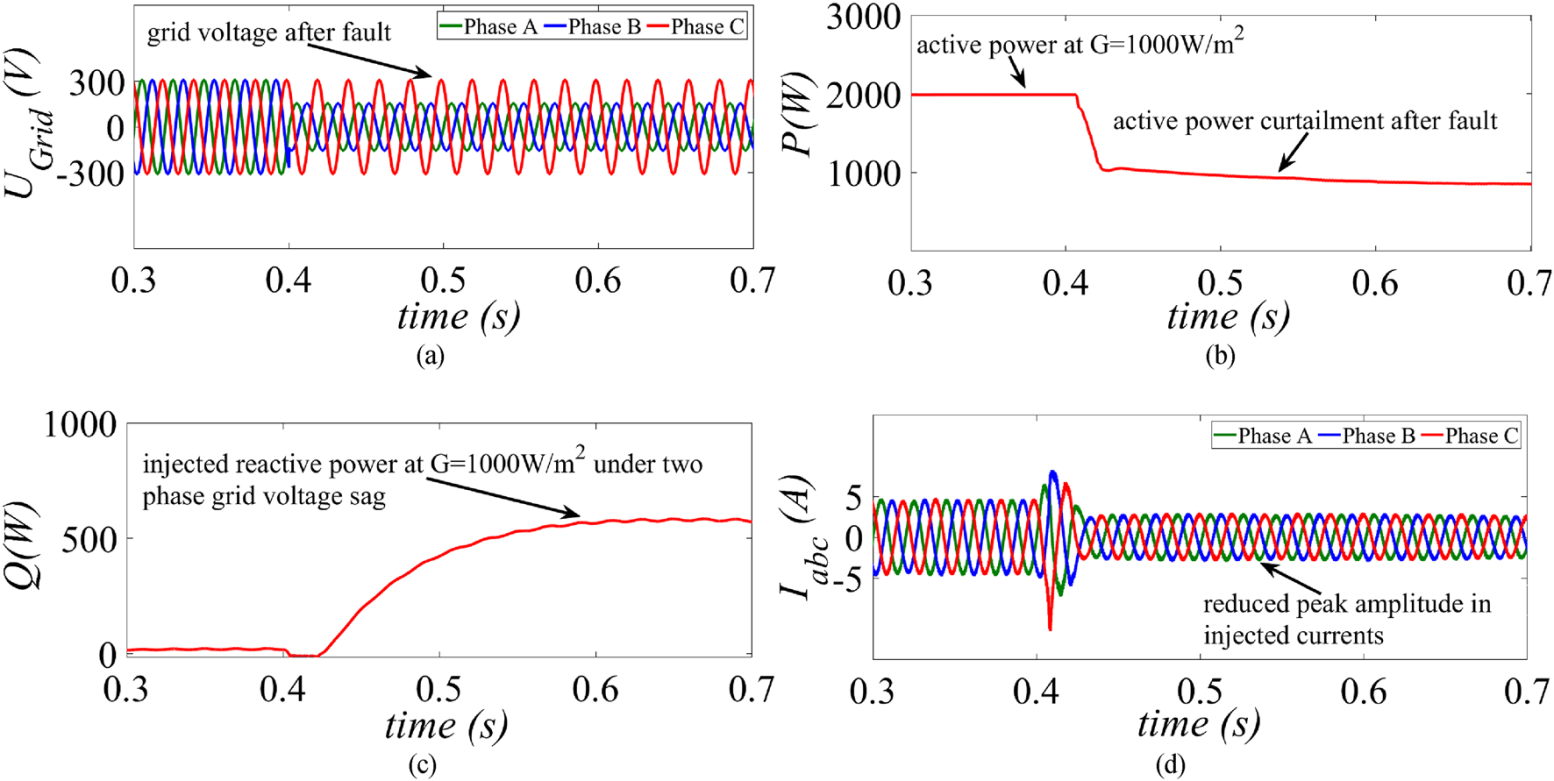
Results of the proposed control strategy for a two-phase grid voltage sag (**a**) grid voltage, (**b**) injected active power, (**c**) injected reactive power and (**d**) inverter current.

The performance of the proposed control strategy is further evaluated under two-phase and three-phase grid voltage sag conditions at standard test operating conditions (G = 1000 W/m^2^ and T = 25 °C). To test the performance under two-phase dip in the grid voltage a fault is created at t = 0.4 s by reducing the phase A and phase B from 1 to 0.2 p.u. as shown in Fig. 11a. The active power is reduced to a safe value of around 1 kW to avoid overcurrent in the inverter current as evident from Fig. 11b. As the magnitude of ${u}_{pu}$ is less as compared to the magnitude in single-phase grid voltage sag condition, a large amount of reactive power around 500 VAr is injected as per the specified grid code as shown in Fig. 11c. As the APC control loop gets activated, the inverter currents remain well below the rated value as shown in Fig. 11d.

**Figure 12:**
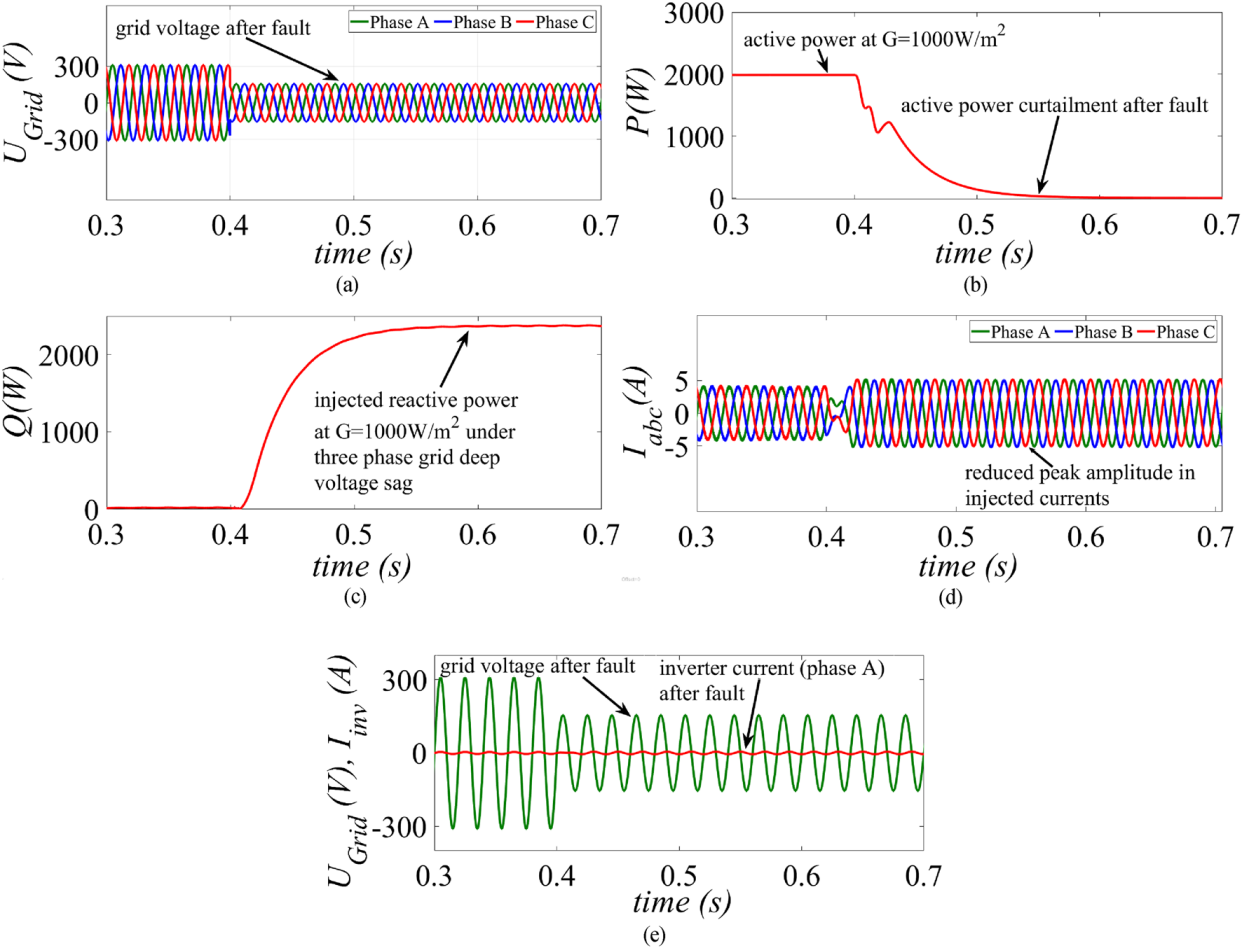
Results of the proposed control strategy for a three-phase grid voltage sag (**a**) grid voltage, (**b**) injected active power, (**c**) injected reactive power and (**d**) inverter current (**e**) Phase-A grid voltage and inverter current before and after fault.

In the second case, the three-phase voltages are reduced from 1 to 0.2 p.u. at t = 0.4 s as shown in Fig. 12a. As there is a severe sag in the grid voltage, the proposed control strategy, completely curtails down the active power and the inverter injects the maximum reactive power at around 2000 VAr to the grid as per the specified grid code as shown in Fig. 12b and c, respectively. Hence, the maximum capacity of the inverter is exploited by injecting balanced sinusoidal reactive currents to the grid as shown in Fig. 12d. To effectively demonstrate this scenario the phase-A grid voltage and phase-A current of the PV inverter is shown in Fig. 12e. The result shows that there is a sag in phase-A grid voltage and is reduced to 0.2 p.u. after t = 0.4 s. On the other hand, the phase-A inverter current is utilized to only inject the reactive power as there is a severe sag in the grid voltage according to the grid code as mentioned in (27). Hence, the proposed strategy assists in supplying the grid with the necessary reactive power and ensures continuous safe operation of the inverter. Moreover, by using the proposed strategy maximum exploitation of the inverter rating is achieved for low, medium and high-power generation condition of GCPV systems.

## Conclusion

This paper presents an improved control strategy that limits overcurrent as well as exploits maximum capacity of a GCPV inverter under unbalanced dip in the grid voltages. The results indicate that the proposed strategy not only reduces the inverter overcurrent to ensure continuous safe operation but also exploits the inverter’s maximum capacity under single, two and three-phase fault conditions. The improved performance of the proposed strategy is established by comparing it with two state-of-the-art control strategies. The main outcomes of the investigations are:

- The results of conventional current reference generation techniques in Fig. 2 indicates that at the expense of oscillating power waveforms, sinusoidal balanced currents can be achieved. Furthermore, the oscillations in active and reactive power can be cancelled out; however, the injected currents then become unbalanced. A control approach that adjusts the current reference vectors can be used to ensure a balanced set of currents injected into the grid with low THD.
- Although the conventional strategies aid in the reduction of power quality issues, they don’t ponder the relative ratio of positive and negative sequence components in reference current during unbalanced grid conditions. A flexible approach for easy regulation of positive and negative current injection is achieved by carefully formulating a control approach by using the two sequence components.
- Under unbalanced grid voltage conditions, the proposed current control technique is used to achieve two objectives; to limit the injected currents and exploitation of inverter’s maximum capacity. The proposed current reference generation technique is formulated in the stationary reference frame and works well under normal as well as faulty gird conditions.
- It can be observed from Fig. 6d, 8d and 10d that under single-phase grid voltage sag, the injected inverter currents remain below the rated inverter capacity and the maximum exploitation of the inverter’s capacity is achieved. At G = 1000 W/m^2^, the proposed strategy provides overcurrent limitation by estimating an expression to determine pseudo inverter capacity (PIC). Moreover, at G = 700 W/m^2^ and 300 W/m^2^, the proposed control strategy has a high-capacity utilization of the inverter power rating.
- The results of the TL-VSS in Fig. 5d shows large inverter overcurrent which will result in the disconnection of the inverter from the grid due to the activation of overcurrent protection device in the inverter. The results of the MR-CLS in Fig. 7d and 9d exhibit low-capacity utilization of the inverter rating.
- The results under two-phase and three-phase dip in the grid voltage shows that the proposed control strategy injects maximum reactive and active power and limits the inverter current by quickly activating the APC control loop during fault-ride-through period. Moreover, under deep voltage sag condition, the active power in is curtailed to zero and the inverter capacity is utilized in injecting the reactive power.

In the near future, the proposed control strategy will be validated on an experimental setup.

## Data Availability

The datasets used and/or analysed during the current study available from the corresponding author on reasonable request.

## Abbreviations

AARC: Average active reactive control
APC: Active power curtailment
BPSC: Balanced positive sequence control
CRG: Current reference generation
DG: Distributed generation
GCPVS: Grid connected photovoltaic systems
IARC: Instantaneous active reactive control
LVRT: Low voltage ride through
MPP: Maximum power point
PCC: Point of common coupling
PIC: Pseudo inverter capacity
PLL: Phase locked loop
PNSC: Positive negative sequence control
RES: Renewable energy resources
SRF: Stationary reference frame
THD: Total harmonic distortion
VSS: Voltage support strategy
VUF: Voltage unbalance factor
VSI: Voltage source inverters
